# Predicting gene expression in T cell differentiation from histone modifications and transcription factor binding affinities by linear mixture models

**DOI:** 10.1186/1471-2105-12-S1-S29

**Published:** 2011-02-15

**Authors:** Ivan G Costa, Helge G Roider, Thais G do Rego, Francisco de AT de Carvalho

**Affiliations:** 1 Center of Informatics, Federal University of Pernambuco, Recife, Brazil; 2 Dept. of Computational Molecular Biology, Max Planck Institute for Molecular Genetics, Berlin, Germany

## Abstract

**Background:**

The differentiation process from stem cells to fully differentiated cell types is controlled by the interplay of chromatin modifications and transcription factor activity. Histone modifications or transcription factors frequently act in a multi-functional manner, with a given DNA motif or histone modification conveying both transcriptional repression and activation depending on its location in the promoter and other regulatory signals surrounding it.

**Results:**

To account for the possible multi functionality of regulatory signals, we model the observed gene expression patterns by a mixture of linear regression models. We apply the approach to identify the underlying histone modifications and transcription factors guiding gene expression of differentiated CD4+ T cells. The method improves the gene expression prediction in relation to the use of a single linear model, as often used by previous approaches. Moreover, it recovered the known role of the modifications H3K4me3 and H3K27me3 in activating cell specific genes and of some transcription factors related to CD4+ T differentiation.

## Background

All cells in a multi-cellular organism arise from the same zygote and thus carry the same genetic information. However, complex regulatory programs allow stem cells to differentiate into distinct cell types. For instance, in response to different infectious agents Naive CD4+ T cells differentiate into at least four types of T helper cells—Th1, Th2, Th17, and inducible regulatory T cells (iTregs) [1]. While all of these cell types are involved in the adaptive immune response they serve distinct roles by secreting different cytokines. For example, Th1 acts against mycobacterial infections by releasing IFNγ, which activates the response of macrophages [1] while Th2 cells secrete various interleukins helping B-cells to induce humoral immunity.

On the transcriptional level, the differentiation process from stem cells to fully differentiated cell types is controlled by the interplay of chromatin modifications and transcription factor activity [2]. Chromatin structure is shaped primarily by histones. The presence or absence of these large globular protein complexes determines the accessibility of the promoter regions for the transcriptional machinery and thus performs a high-level control on gene expression [3,4]. The affinity of histones to DNA is modified by the cell via a large repertoire of post-translational protein modifications including acetylations and methylations.

The resulting epigenetic histone code appears highly intricate, with a given histone frequently carrying several different modifications at a time. Despite this complexity, it has become clear that certain modifications, such as the trimethylation of the lysine 4 residue in the tail of histone H3 (abbreviated H3K4me3) are mainly associated with active promoters while other modifications such as H3K27me3 tend to be associated with inactive promoters [5]. The importance of histone modifications for the differentiation of Naive CD4 T-cells into Th1 cells has recently been verified at [6], which demonstrated that IFNγ expression is controlled by the histone methylation status of its promoter.

Aside from chromatin structure, transcription factors (TFs), play an essential role in controlling cell differentiation by guiding the transcriptional machinery to its target promoters and facilitating the initiation of transcription. For instance, in T-cell differentiation, in vitro studies demonstrated that either high levels of the transcription factor GATA3 or strong signalling via the transcription factor STAT5 is sufficient to determine the Th2 cell fate [1].

Particularly in the context of genome wide studies, computational biology analysis have become an essential component of elucidating the regulatory signals underlying observed gene expression patterns. Usually, the problem of identifying the promoter elements guiding differentiation and cell type specific gene expression is tackled by first selecting the genes which are most specifically expressed in the particular cell type and then performing motif over-representation analysis on their promoter sequences as in [7,8] (see [9] for a recent review). While such methods allow identifying potentially regulating transcription factors they have the intrinsic drawback of requiring a previous grouping of genes and of being able to explain only the expression of the genes with highest specificity for the condition.

In contrast, linear regression models, as first proposed by [10,11], combine all regulatory signals in order to explain the expression pattern of the genes. In their work, Bussemaker *e*t al. [10] focused on explaining gene expression based on combinations of predicted TF binding sites. The coefficients of the linear model indicate the importance of a particular regulatory signal. That is, signals which obtain large positive coefficients likely correspond to putative activators while signals with large negative coefficients likely act as suppressors. Recently, Karlic *e*t al. [12] also used a linear regression model in order to estimate promoter activity based on histone modification data. By design, the above approaches assume that a given regulatory signal exert the same regulatory effect on all its target genes.

However, transcription factors and thus their DNA binding motifs frequently act in a bi-functional manner, with a given DNA motif conveying both transcriptional repression and activation depending on its location with respect to the transcription start site (TSS) and the sequence motifs surrounding it. For instance, RUNX1 and RUNX3 have been shown to act both as repressors and activators in different tissues and are involved in determining T-cell fate [13].

**Figure 1:**
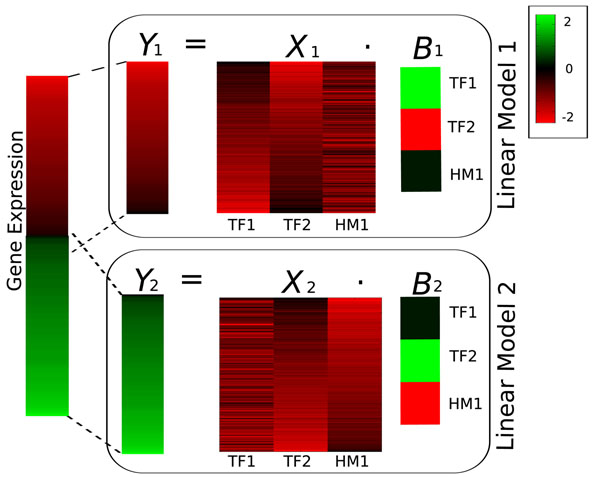
**Example of Linear Regression Mixture Model**. Illustrative example of the use of linear regression mixture models to predict gene expression from the regulatory signals. Gene expression profiles, indicated by the heat map on the left (red and green correspond to highly and lowly expressed genes, respectively), are best predicted by two different models: model 1 for genes with high to medium expression and model 2 for genes with medium to low expression. At the level of each linear model *k*, the gene expression vector *Y_k_* is predicted by the multiplication of the matrix *X_k_* (bright red values indicate higher TF binding strength or HM presence) with the vector of model coefficients *B_k_* (red, black and green values indicate positive, near zero and negative regression coefficients, respectively). In this example, *B*_1_ indicates that genes with high to medium expression are repressed by TF1, positively regulated by TF2 and are unrelated to the presence of HM1. In contrast, *B*_2_ indicates that genes with medium to low expression are repressed by TF2, positively regulated by HM1, and are unrelated to the binding affinity of TF1. If we estimate a single linear model from the same data, TF2 would have a regression coefficient close to zero as the positive coefficient from model 2 and the negative coefficient from model 1 would cancel each other out.

To account for the possible multi functionality of regulatory signals, in this study, we propose to extend [10-12] by allowing the observed gene expression patterns to be explained by not just one, but by a mixture of several linear regression models [14,15]. This permits for instance to find mixture models, such that genes with high maximal expression are controlled by a different group of regulatory signals than genes with low maximal expression (see Fig. 1). That is, a regulatory signal might act as repressor when associated with lowly expressed genes while it may function as activator or neutral bystander when present in the promoter of highly expressed genes.

In order to find the regression models best explaining the expression data, our method takes as input the matrix *Y* of observed expression profiles from all genes as well as a matrix *X*, containing the regulatory signals for the corresponding promoters (i.e. predicted TF binding affinities and presence of histone modifications). For each gene, it then estimates the coefficient vector *B*, representing the relative importance of each regulatory signal and its effect on gene expression (activation or repression).

We apply this novel approach to identify the underlying regulatory signals guiding gene expression in each of the four differentiated CD4+ T-helper cell types. As potential regulatory signals we consider both, histone modifications (HM) as measured by Chip-Seq [16] as well as predicted binding affinities [17] from a set of TFs related to lymphoiesis [1,18-20]. As we are mainly interested in cell type specific signals, we restrict the analysis to genes with low CpG content in their promoters [21] as such genes tend to be expressed in a tissue and stage specific manner while genes with high CpG promoter content tend to be broadly expressed. Using this method we expect to improve the gene expression prediction in relation to the use of single linear model, but also to reveal the regulatory roles for histone modifications and transcription factors.

## Results and discussion

### Regulatory signals predicts expression

As a first step, we want to determine which set of regulatory signals, *X*, can explain the observed gene expression data, *Y* , best. To this end, as a first step we supply our algorithm with a matrix *X* containing only predicted TF binding affinities, only histone modification data or both sets of regulatory signals and assess how well the resulting regression models can capture the data. As measure of quality for the different models we thereby compute the mean square error between the predicted gene expression values and the actual measurements (see Methods for details).

**Figure 2:**
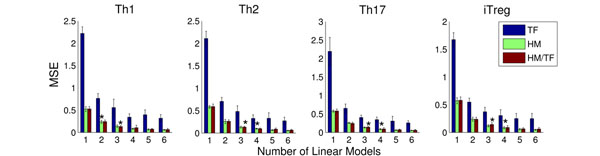
**Regression Prediction Error.** We depict the MSE error for 1 to 6 models for the prediction of expression on Th1, Th2, Th17 and iTreg. Bars marked with * indicate number of linear models indicated by the model selection. The MSE with TF is higher than the use of either HM/TF or HM on all combinations of expression data and number of models. For all combination of expression data and number of models, the was no significant difference between the MSE from HM or HM/TF.

Predicting the gene expression from the four T-cell types based on only histone modification data and by means of only a single regression model yields MSEs of about 0.5 for HM and HM+TF on all data sets (see red bars in Fig. 2). A mixture of two regression models further reduces the MSEs to an average value 0.25 across all cell types. In all scenarios, the difference of MSE between one and two models were statistically relevant (*t*-test *p*-value < 0.01) indicating the advantage of using mixtures to predict expression. The model selection procedure (see Methods) indicates that the data is optimally explained by the combination of 2-4 regression models (see Fig. 2) and that gene expression data can be well predicted based on histone modification data alone.

In contrast, using a single regression model to predict gene expression data based on TF binding affinities alone yields considerably larger MSEs across all cell types (average MSE = 2, see blue bars in Fig. 2). Interestingly, supplying our algorithm with the combined data from both histone modifications and TF binding affinities yields MSEs similar to the ones obtained with only histone modification data alone (see Fig. 2). This indicates that the utilized histone modification and TF binding data cover rather redundant than complementary information about gene expression. As histone modifications and HM+TF affinities yield the solutions with the lowest MSEs, in the following we will continue the analysis with the models based on these data sets.

### Control of Th1 gene expression

Having established that histone modification data together with a mixture of two regression models yields the most significant results we now investigate which type of modifications contribute most strongly to these models. We thereby restrict our analysis to the data from Th1 cells and the corresponding regulatory signals which obtain the largest absolute regression coefficients (results from other cell types closely resemble those from Th1 cells, see Additional File 1 for details).

**Figure 3:**
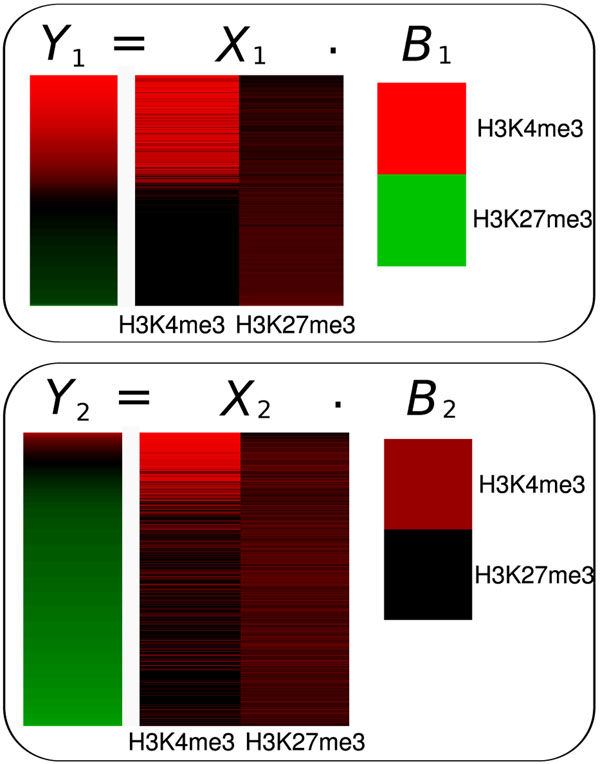
**Regression Results Th1**. Results for mixtures of two regression models utilizing only histone modification data on Th1. Model 1 captures the expression of 2231 genes and model 2 of 3923 genes. Corresponding gene expression levels are shown by the vectors Y on the left (red and green indicate high and low expression, respectively). Association strengths of the regulatory signals for the corresponding promotes are indicated by matrices X (red and black indicate high and low association, respectively). Finally, the magnitude of the corresponding regression coefficients for each of the regulatory signals in each of the models is indicated by vector B (bright red for large positive and bright green for large negative values). In model 1, the amount of H3K4me3 correlates positively while the level of H3K27me3 correlates negatively with gene expression. In model 2, only H3K4me3 levels correlate with gene expression as H3K27me3 levels remain constant over the whole expression range.

For model 1, which explains the expression pattern of the most highly as well as moderately expressed genes, the histone modifications with largest influence are H3K4me3 and H3K27me3, with regression coefficients of +0.7975 and -0.4533, respectively. As shown in the top part of Fig. 3 these two modification form a gradient with H3K4me3 being most frequently found in the highly expressed genes while being absent in moderately to lowly expressed genes. In contrast, H3K27me3 is consistently detected in promoters of lowly expressed genes but appears weaker or even absent in promoters of highly expressed genes.

For model 2, which explains the transcriptional activity of a small subset of highly expressed as well as most of the lowly expressed genes, we again find H3K4me3 to have the strongest positive regression coefficient (*b* = 0.71). This is reflected by a strong association of this modification with the most highly expressed genes of this set (see Fig. 3). In contrast, H3K27me3 obtains a regression coefficient of close to zero (*b* = 0.03) in this model as this modification appears with the same intensity in nearly all genes assigned to model 2.

**Figure 4:**
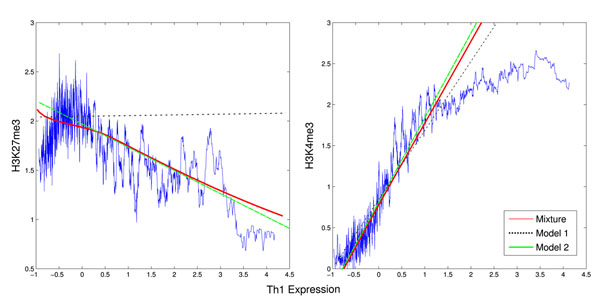
**Histone Modification against Th1 Gene Expression**. We depict the values of Th1 gene expression against H3K27me3 modification (left) and H3K4me3 modification (right). The blue line represents a nearest-neighbor interpolation (30 samples) of the histone modification signal, the dashed black line represents the linear model for the 1 component, the green line for the 2 component and the red curve represents the mean regression value of the mixture of linear regression with 2 components. The interpolation indicates that the H3K4me3 is close to linear, but not for H3K27me3 modification, which is negatively related to high expressed genes, but has little effect on low expressed genes.

**Figure 5:**
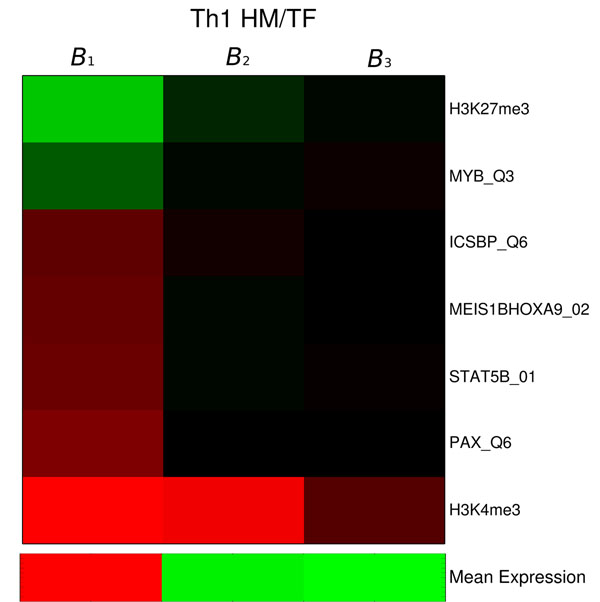
**Regression Coefficients on Th1 with HM/TF data.** We depict the regression coefficients of the most relevant regulatory signals and mean expression values for the mixture with three linear models on Th1 with HM/TF data.

An alternative view of this results is presented at Fig. 4. There, we have the interpolated values of the histone modifications against the gene expression, the linear models for each component and the resulting mixture model. Clearly, dependence of H3K27me3 on gene expression is not linear, as low expressed genes all present a high presence of histone modifications. This non-linearity is captured by the mixture model (red line), and explains the lowest MSE errors obtained when more than one linear models is applied. To see whether the influence of TFs may contribute to this effect we next look in detail at the results obtained from combined histone and TF data together with a mixture of three correlation models. As shown in Fig. 5), we see similar results in respect to the histone markers: H3K4me3 as enhancer and H3K27me3 as inhibitor of expression for genes with high expression and H3K4me3 as enhancer for genes with low expression. Moreover, only for the genes with high expression, there are some TFs (Pax5, Stat5, Meis/Hox, Iscbp) promoting gene expression and a TF (MyB) inhibiting expression. For all TFs, regression coefficients were in the range of 0.1 to 0.15 (see Additional File 1 for additional results). For genes with low expression, we found no relation between the TFs binding affinities and expression. Th1 cells are known to be regulated by T-bet and Stat4 [1]. While our study lacked the PFM of T-bet, it listed the closely related Stat5 as a positive regulator of the genes with high expression. In relation to factor related to inhibition, there has been a recent implication of c-MyB to bind to H3 histone tails and to promote histone acetylations in Humans [22]. These results indicate a putative role of the MyB in down-regulating the expression of genes during CD4+ T differentiation by promotion of epigenetic changes. However, further acytilation modification data would be required for a better characterizaion of the role of this factor.

### Comparison with previous studies

Several computational biology methods have been previously proposed for the use of linear models for predicting gene expression in the context transcription factor binding [10,11] or histone modifications [12]. In all cases, distinct datasets were used and results are not directly comparable. In relation to [12], the analysis were based on Human naive CD4+ T cells and included 38 histone modifications. Their predicted model obtained a correlation coefficient of 0.72 on genes with low CpG content with HM H3K4me3 and H3K79me1, while our method had a coefficient at the range 0.64 – 0.68 for one model and 0.85 – 0.87 for two models for H3K4me3 and H3K27me3 data. The increase of the correlation coefficient from single linear models to two linear models is an indication that all these approaches would profit from the use of the mixture of linear regressions framework.

## Conclusion

Predicting gene expression from regulatory signals is an important but unmet goal in bioinformatics. In this study, we propose a novel approach which uses mixtures of linear regression models together with transcription factor binding and histone modification data for estimating transcriptional activity of CpG depleted promoters. In addition the approach allows to determine the functional activity of the various regulatory signals. We show that our approach obtains significantly smaller errors in predicting the expression of genes in comparison to simple linear regression models as used in previous approaches. For gene expression data from CD4+ T helper cells we find that both, histone modification data alone and histone modifications together with predicted TF binding affinities, yields the best expression predictors. In accordance with previous dedicated studies we recover the well known regulatory roles of H3K4me3 as an enhancing and H3K27me3 as a repressive regulatory signal for gene expression. Moreover, our predictions suggest that histone modifications act not in a binary on/off fashion but rather in a continuous way with levels of H3K4me3 and H3K27me3 steadily rising or falling over a large range of expression values in a non-linear way. With the use of TF binding affinities, we also partially recover the main factors such as the Stat family involved in T helper cell type specific gene expression. Interestingly, we observe a negative effect of cMyb on expression in all T helper cell types. This raises the question whether MyB, which has been recently showed to promote histone acetylation marks in hematopoiesis [22], could play a role in the down-regulation of genes in T helper cells types.

The advent of next generation sequencing provides an ever growing stock of high quality data for the full range of histone modifications, DNA methylation state and transcription factor occupancy across the entire genome from various cell types and differentiation stages. Several methodological improvements will be required to integrate this wealth of data in order to shed light on the complex interplay between the different regulatory signals acting in eukaryotics. Moreover, in an ideal case where all possible regulatory signals have been measured, advanced feature selection procedures such as postulated by [23], will be vital for the detection of all the players involved in determining gene expression.

## Methods

### Mixture of linear regressions

In the following we want to model the observed expression level of all *N* genes, using different linear combinations of the *M* different regulatory signals associated with the promoters (i.e. binding affinities for various TFs and different histone modifications). To this end let *y_i_* be the gene expression level of gene *i* (the dependent variable) and *x_i_* be a corresponding vector of *M* regulatory signals (the regressor variables). The single linear regression model is then defined as

*y_i_=b*_0_*+ x_i_B***^T^** + *∊*_i_, (1)

where *B* is a vector (*b*_1_, …,*b_M_*) representing regression coefficients and *∊*_i_ is an error term. For mathematical convenience, we redefine the vector with the regressor variables to be *x_i_* = (1,*x_i_*_1_, …,*x_iM_*) and include the bias parameter *b*_0_ in the beginning of *B*, that is *B =* (*b*_0_, *b*_1_,…, *b_M_*). Assuming the error *∊* follows a Normal distribution with standard deviation *σ*^2^, the linear regression model has the following distribution

ℙ(*y_i_|x_i_*, *B*,*σ*^2^ ) *=***N**(*y_i_|x_i_B***^T^**,σ^2^ ). (2)

A mixture of linear regression models is defined as a convex summation of *K* distributions(3)

where Π = (*π*_1_, …,*π_K_*) are the mixture coefficients, which respect *π_k_* ≥ 0 and  , and Θ are the model parameters (Π, *B*_1_,…, *B_K_*, , …, ).

For a given data *X* and *Y*, where *X* is a set on *N* observations *x_i_* and *Y* a vector with *N* observations *y_i_*, the mixture of linear regression models can be estimated with the Expectation-Maximization algorithm [14,24]. We resort to Maximum-a-posteriori (MAP) estimates of the parameters, as described in the next section, to avoid over-fitting [25]. The EM works by finding estimates Θ maximizing the posterior distribution over the data *X* and *Y*

ℙ(Θ|*X*, *Y*, *Z*) ≈ ℙ(*Y*, *Z*|*X*,Θ)ℙ(Θ) (4)

where *Z* is the vector of hidden variables with *z_i_* ∈ {1,…, *K*} indicating which linear model an observation *i* belongs to. ℙ(Θ) is the prior distribution over the model parameters (see next section for the definition of the prior). ℙ(*Y*, *Z|X*, Θ) is the complete data likelihood and is given by:(5)

where *r_ik_* is the posterior probability (or responsibility) [25] that observation *i* belongs to the linear model *k* and is given by:(6)

For further details on mixture models we refer the reader to [25].

The EM algorithm works by iteratively estimating the model assignments (*r_ik_*) and the model parameters Θ until some convergence criteria is reached. In the context of the mixture of linear regression models, we need estimates of the linear regression parameters (*B_k_*, ) for a particular model *k*, and all other parameters (*r_ik_*,Π) follow the usual EM algorithm [25].

Once the mixture model is estimated, the predicted value *ŷ_i_* for a particular regressor observation *x_i_* is given by(7)

That is, the linear regression prediction is a mixture of the predictions of each individual component times the posterior probability of the observation *i* to belong to the model *k.* In our particular application problem, we are interested in estimating the models which corresponds to an unsupervised learning problem, that is, the coefficients indicating whether a regulatory signal plays an important repressive or activating role. The predictions *ŷ* can thereby be used for evaluating the fit of our model. In cases where one wants estimate the expression level of genes, that is, estimation of *ŷ* (supervised learning problem), the above equation should not be used, as the posterior probabilities are based on the response variable *y*, which is usually unknown in a predictive scenario. In such a context, methods for combinations of predictors, such as [26], are required.

### Bayesian linear regression estimates

We resort to Bayesian approach for obtaining MAP estimates of the linear regression models as proposed in [27]. Therefore, we avoid problems related to over-fitting which usually occur with the EM algorithm and mixture models [25]. More formally, the prior distribution in Eq. 4 can be decomposed as(8)

We use the following conjugate prior for the regression coefficient *B_k_*

ℙ(*B_k_*) = ***N***(*B_k_*|0, *β_k_***I**), (9)

where 0 is a vector with *M* zeros, **I** is a *M* x *M* identity matrix and *β_k_* is the hyper-parameter.

Let *r_k_* be an *N* dimensional vector (*r*_1_*_k_*, …,*r_Nk_*) containing the posterior probabilities of the observations belonging to model *k* and let *W_k_ =* diag(*r_k_*), then the estimates from model *k* maximizing Eq. 4 are defined as(10)

with(11)

From Eq. 10, we can see that *β_k_* works by shrinking the regression coefficients. Small *β_k_* imposes a higher shrinkage on the regression coefficients. Furthermore, for *β_k_* → ∞ we have a non-informative prior and the regression coefficients are the maximum likelihood estimates.

We estimate the hyper-parameter *β_k_* in an Empirical Bayes approach with(12)

where(13)

and *λ_j_* is the *jth* eigenvalue of the PCA decomposition of matrix  (see [27] for details).

Note that *β_k_* requires the definition of *B_k_*, which in our context is taken from the previous iteration of the EM algorithm.

For the mixture mixing coefficients, we use a symmetric Dirichlet distribution as prior

ℙ(Π) = Dirichlet(Π|α), (14)

where *α* is the hyper-parameter. Hence, the mixing coefficients estimates used by the EM algorithm are(15)

We use a prior of *α =* 2, which avoids models with a low number of observations assigned to it.

### Transcription factor affinity

TF binding motifs are traditionally described in the form of position frequency matrices (PFMs). PFMs show how often a certain base occurs at a given position in the alignments of known binding sites of the TF. To predict the binding strength of a given TF to a promoter sequences we utilize the TRAP method [17]. In contrast to motif matching algorithms which make a binary distinction between binding sites and non-binding sites, TRAP avoids this artificial separation and instead computes the probability of a TF to bind site *i* in the sequence using the following equation(16)

where *δE_i_*(*λ*) is the energy difference between the state in which the factor is bound to site *i* and the state in which the factor is bound to its consensus site. This so called mismatch energy is scaled by a parameter *λ* which was previously determined to have an optimal value of 0.7 [17]. The second transcription factor dependent parameter *R*_0_ determines both, the binding energy between the factor and its consensus site as well as the TF concentration. *R*_0_ is derived for each PFM individually as

*R*_0_ = exp(0.6 • *W* – 6), (17)

where *W* is the number of columns in the PFM with information content exceeding 0.1 bits. Matrix positions which fall below this entropy cutoff also do not contribute to the mismatch energy in Eq. 16. The nucleotide dependent mismatch energies for each site in the promoter sequence are computed by(18)

where *v_i_*_,_*_max_* is the frequency of the consensus base at position *i* in the PFM and *v_i_*_,_*_α_*, is the frequency of the observed base *α* at position *i* in the PFM. Eventually, TRAP obtains the expected number *N* of TFs bound to the promoter by summing over the individual probabilities from all *L* sites in the sequence:(19)

As input, TRAP requires for each TF a PFM suitable for computing the mismatch energies and a DNA sequence of interest (see [17] for details).

For our study we use a selection of 102 PFMs from the Transfac database version 11.1 [28], which correspond to TFs involved in lymphoid development (see Additional File 1 for TF list). As we are mainly interested in binding sites near the promoter, the analysis was based on the 200 base pairs upstream of the transcription start site (TSS) of the genes. We restrict the analysis to genes with normalized CpG content < 0.5 in their promoter sequence [21], as such genes tends to be expressed in a tissue and stage specific way. In the end, we calculate the affinity (Eq. 19) for all the selected genes and PFMs. This yields the matrix *X* containing the TF binding data, where *x_i_*_,_*_j_* corresponds to the affinity of TF *j* to the promoter of gene *i.*

### T-cell gene expression and histone modification data

We use the gene expression and histone modification data from Th1, Th2, Th17 and iTreg cells published by [16]. The histone modification data was measure with the Chip-Seq Illumina platform. We used the Cisgenome tool [29] to align sequence data and to detect peaks. As we are only interested in the modifications near the promoter, we consider the region of 8000 bps upstream and 2000 bps downstream of the TSS and kept the tag counts of the highest peak. Finally, we added a pseudo count to avoid zero values and applied a log transform. This yields the matrix *X* containing the histone modification data, where *x_i_*_,_*_j_* corresponds to the number of ChIP-seq tags derived from a particular histone modification *j* that are being mapped to the promoter of gene *i.*

The expression data was measured with Affymetrix 430 chips. The raw data has been normalized using the variance stabilization method of [30] and normalized the tissues to have mean expression equal to zero.Microarray probes were mapped to ENSEMBL gene identifiers with the help of the biomart tool [31]. We thereby kept all genes that had their expression measured by multiple probe sets. In the following, we restrict our analysis to those 6154 genes with low CpG content for which both, gene expression as well as histone modification data is available. The final data sets used in this analysis can be found at http://www.cin.ufpe.br/~igcf/MixLin.

### Experimental design

We model gene expression from four different T helper cell types (Th1, Th2, Th17 and iTreg) with the use of either transcription factor affinities (TF), histone modifications (HM) or both regulatory signals combined (HM+TF). As parameter of our method, we vary the number of linear models, *K*, from 1 to 6. In order to select the optimal model for each cell type, we first perform 10 fold cross-validation on each parameter setting and then estimate the Mean Square Errors (MSE) from the validation sets. As the MSE tends to decrease with higher *K*[32], we use a model selection procedure, the Bayesian Information Criteriun (BIC) [25], to indicate the optimal number of models. The method has been implemented with Pymix [33] and is freely available at http://www.pymix.org.

## Authors contributions

IGC, RH, TGR implemented the approach and performed the experiments. IGC, RH and FATC designed the study and evaluated the results. All authors wrote the manuscript. All authors read and approved the final manuscript.

## Competing interests

The authors declare that they have no competing interests.

## Supplementary Material

Additional file 1 **Supplementary Figures and Tables** This file contains additional Figures and Tables. Click here for file
