# Supplementary material for: Predicting gene expression in T cell differentiation from histone modifications and transcription factor binding affinities by linear mixture models
Source: BMC Bioinformatics. 2011 Feb 15;12(Suppl 1):S29. doi: 10.1186/1471-2105-12-S1-S29 (PMC3044284; doi:10.1186/1471-2105-12-S1-S29)
Supplement: Additional file 1 — Supplementary Figures and Tables This file contains additional Figures and Tables. [file 1471-2105-12-S1-S29-S1.pdf]

Predicting Gene Expression in T cell Differentiation from Histone Modifications and Transcription Factor Binding Affinities by Linear Mixture Models

Ivan G. Costa*[1] , Helge G. Roider,[2], Thais G. do Rego,[1] and Francisco de A. T. de Carvalho [1]

[1]Center of Informatics, Federal University of Pernambuco, Recife, Brazil

[2]Dept. of Computational Molecular Biology, Max Planck Institute for Molecular Genetics, Berlin, Germany

Email: Ivan G. Costa*- igcf@cin.ufpe.br;

*Corresponding author

1 Supplemement

Gene Name	MGI Symbol	Function/Cell Type	Reference
PU.1	Trim14	Myeloid Commit.	[19]
LyF-1	Ikaros/Zfpn1a1	Lymphoid Commit.	[19]
Ik-3	Ikaros/Zfpn1a1	Lymphoid Commit.	[19]
Aiolos	Zfpn1a3	Immt. B Commit	[19]
E12	E21,Tcfe2a	Pro-B Commit	[19]
Olf-1	Ebf	Pro-B Commit	[19]
OCA-B		Mature B	[19]
PAX-5	Pax5	Pro-B Commit	[19]
LEF-1	Lef1	Pro-B Commit	[19]
SOX4	SOX4	Pro-B Commit	[19]
IRF-4	Irf4	Pre-B Commit	[19]
POU2F1	OCT-1/Slc22a1	Immat. B	[19]
POU2F2	OCT-2/Slc22a2	Germ. B	[19]
REL-A	Rela		[19]
c-REL	Rel		[19]
p50	Nfkb1	Spleen B	[19]
Notch	Notch1	T-Cell	[19]
CBF1	RBP-Jk,Rbpsuh		[19]
Id2	Id2	Pro-NK	[19]
IRF-8	IRF8	Pre-B	[18]
Stat4	Stat4	Helper T2	[1]
Stat6	Stat6	Helper T2	[1]
C/EBPbeta	Cebpb	Bcell	[18]
C/EBPgamma	Cebpg	Bcell	[18]
PLZF	Zbtb16	Early Lymp.	[18]
HOXA10	Hoxa10	Ealry Hemap./ Down in B-cell	[18]
HOXA9	Hoxa9	Down Reg. in B-Cell TCell	[18]
HOXB3	HoxB3		[18]
HOXB4	HoxB4	HSC	[18]
c-MYb	myb	EHP	[18]
GATA-3	Gata3	NK-TCell	[18]
Stat3	Stat3	IL-6 Signalling [18]	[18]
SCL	Ccl21b	HSC	[18]
IRF-1	Irf1	Selection of CD8+ T Cells	[18]
Pax-5	BSAP	Pro-B	[18]
HEBalt	Tcf12	tcell	[2]
Bcl11b	Bcl11b	tcell	[20]
HEBcan	Tcf12	tcell	[20]
Tcf-1	Tcf7	tcell	[20]
c-Ets-1	Ets1	tcell	[20]
Id3	Id3	tcell	[20]
Lef-1	Lef1	tcell	[20]
c-Ets-2	Ets2	tcell	[20]
Spi-B	SpiB	tcell	[20]
Hes-1	Hes1	tcell	[20]
Sox13	Sox13	tcell	[20]
Gfi1	Gfi1	tcell	[20]
AML1	Runx1	tcell	[20]
Gata-3	Gata3	tcell	[20]
E12	E2A,Tcfe2a	tcell	[20]
RunX3	Runx3	tcell	[20]
Id2	Id2	tcell	[20]
PU.1	Sfpi	tcell	[20]
Scl	Tal1	tcell	[20]
Gata-2	Gata2	tcell	[20]
C/EBPalpha	Cebpa	tcell	[20]
NFAT		tcd4	[1]
Stat1		tcd4	[1]
T-bet		tcd4	[1]
c-MAF	MAF	tcd4	[1]
AP1	AP-1	tcd4	[1]
FOX3P		tcd4	[1]
RORalpha		tcd4	[1]
RORgamma		tcd4	[1]
RUNX1		tcd4	[1]
Stat5		tcd4	[1]

Table 1: List of transcription factors related to Lymphoid development.

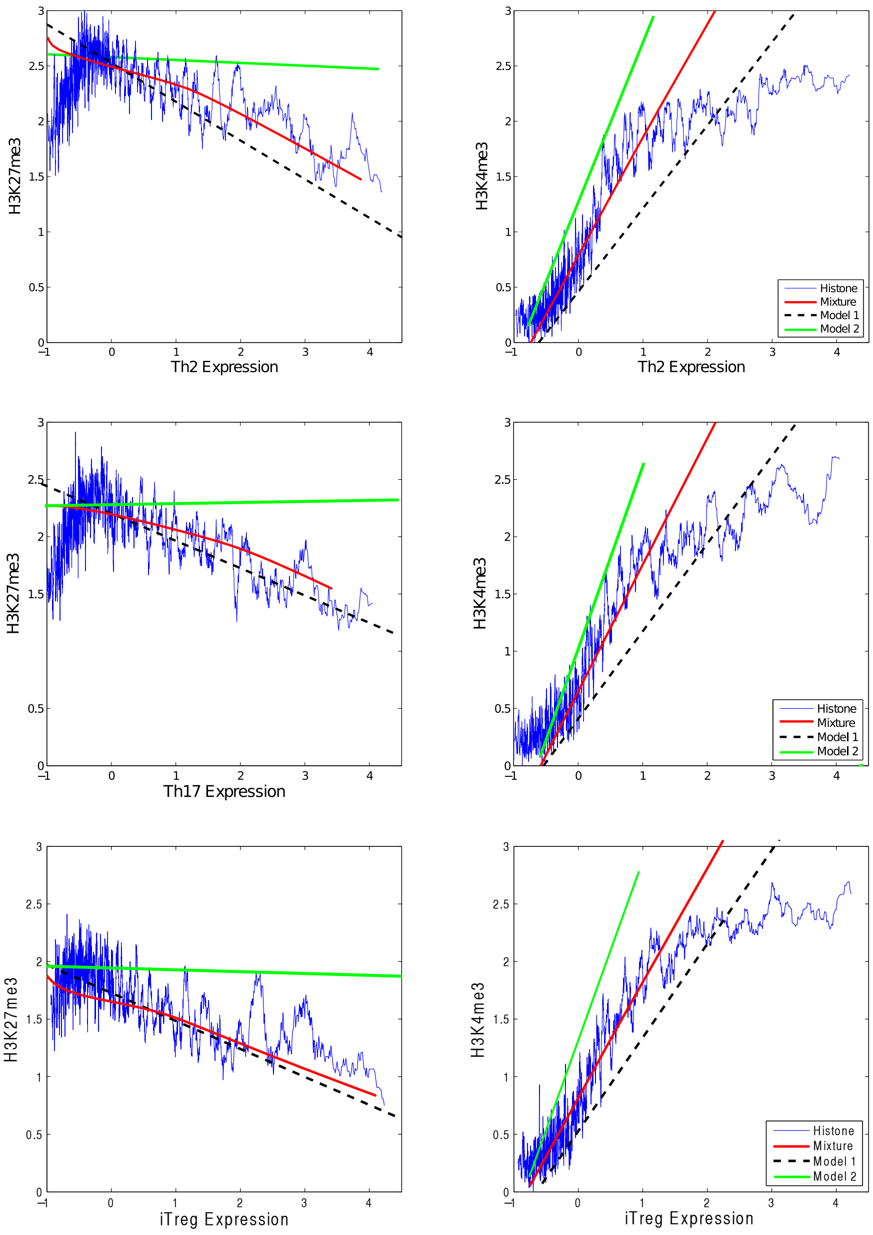

Figure 1: We depict the values of Th2/Th17/iTreg gene expression against H3K27me3 modification (left) and H3K4me3 modification (right). The blue line represents a nearest-neighbor interpolation (30 samples) of the histone modification signal, the dashed black line represents the linear model for the 1 component, the green line for the 2 component and the red curve represents the mean regression value of the mixture of linear regression with 2 components.

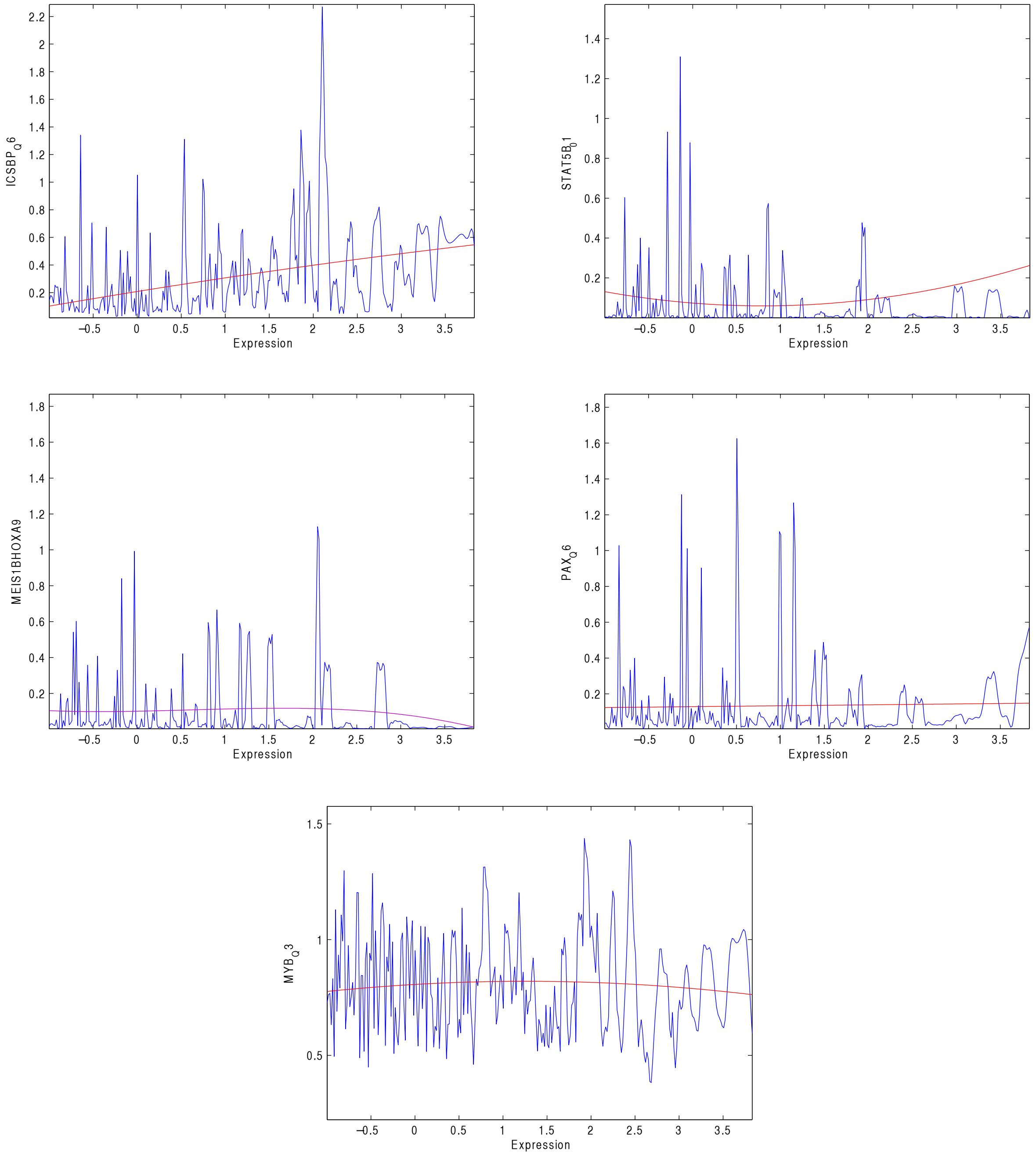

Figure 2: We depict the values of Th1 gene expression against binding affinities of Iscbp, Meis1/Hoxa9, MyB, Pax5 and Stat5. The blue line represents a nearest-neighbor interpolation (30 samples) of the transcription factor affinity signal, the red curve represents the mean regression value of the mixture of linear regression with 3 components. As indicated in Fig 5 of the main manuscript, there is a small tendency of higher affinities of Iscbp, Meis1Hoxa9,Pax5b and Stat5 for genes with high expression, while there is a small tendency for lower cMyb affinities for genes with high expression.
